# Hypertension and coronary artery ectasia: a systematic review and meta-analysis study

**DOI:** 10.1186/s40885-021-00170-6

**Published:** 2021-07-15

**Authors:** Mostafa Bahremand, Ehsan Zereshki, Behzad Karami Matin, Mansour Rezaei, Hamidreza Omrani

**Affiliations:** 1 Cardiovascular Research Center, Health Institute, Kermanshah University of Medical Sciences, Kermanshah, Iran; 2 Research Center for Environmental Determinants of Health (RCEDH), Health Institute, Kermanshah University of Medical Sciences, Kermanshah, Iran; 3 Imam Reza Hospital Research Center, Kermanshah University of Medical Sciences, Kermanshah, Iran

**Keywords:** Hypertension, Coronary artery disease, Meta-analysis, Systematic review

## Abstract

**Background:**

Coronary artery ectasia (CAE) is characterized by the enlargement of a coronary artery to 1.5 times or more than other non-ectasia parts of the vessel. It is important to investigate the association of different factors and CAE because there are controversial results between available studies. We perform this systematic review and meta-analysis to evaluate the effects of hypertension (HTN) on CAE.

**Methods:**

To find the potentially relevant records, the electronic databases, including Scopus, PubMed, and Science Direct were searched on 25 July 2019 by two of the authors independently. In the present study, the pooled odds ratio (OR) accompanied by 95 % confidence intervals (CIs) were calculated by a random-effects model. Heterogeneity presented with the I^2^ index. Subgroup analysis and sensitivity analysis by the Jackknife approach was performed.

**Results:**

Forty studies with 3,263 cases and 7,784 controls that investigated the association between HTN and CAE were included. The pooled unadjusted OR of CAE in subjects with HTN in comparison by subjects without HTN was estimated 1.44 (95 % CI, 1.24 to 1.68) with moderate heterogeneity (I^2^ = 41 %, Cochran’s Q *P* = 0.004). There was no evidence of publication bias in the analysis of HTN and CAE with Egger’s test (*P* = 0.171), Begg’s test (*P* = 0.179). Nine articles reported the adjusted effect of HTN on CAE by 624 cases and 628 controls. The findings indicated the overall adjusted OR was 1.03 (95 % CI, 0.80 to 1.25) with high heterogeneity (I^2^ = 58.5 %, Cochran’s Q *P* = 0.013).

**Conclusions:**

We found that when the vessel was in normal condition, HTN was not very effective in increasing the chance of CAE and only increased the CAE chance by 3 %. This is an important issue and a warning to people who have multiple risk factors together. More studies need to be performed to further establish these associations by reported adjusted effects.

**Supplementary Information:**

The online version contains supplementary material available at 10.1186/s40885-021-00170-6.

## Background

Coronary artery ectasia (CAE) is characterized by the enlargement of a coronary artery to 1.5 times or more than other non-ectasia parts of the vessel [1]. CAE is defined by increasing the pressure of the wall vessel, the thin arterial wall which causes advanced dilation and reforming of the vessel [2]. The fixed dilation of the artery is to be usually caused by inflammation, disease, chemicals, or physical stress of the vessel [2]. It makes the heart tissue to be deprived of blood and die because of decreased blood flow and blockages due to blood clots or spasms of the blood vessel [3]. CAE is commonly asymptomatic and is normally discovered when performing tests for other conditions such as coronary artery disease, stable angina, and other acute coronary syndromes. It is estimated that the incidence of CAE was to be in the range of 1–5 % in angiographic examinations [4]. Some studies had indicated the risk factors such as; hyperlipidemia, obesity, diabetes mellitus (DM), and other factors that could be significantly associated with a higher risk of CAE [5–11]. The relationship between CAE and hypertension (HTN) is not clear in the view of pathophysiology, or cause and result but it is commonly found in patients with diseases of atherosclerosis, connective tissue, and an increased inflammatory response [12, 13]. Also one of the clinical reasons for CAE might be the pressure effect on the artery wall in the blood flow. It results in the artery dilate by pushing blood on the artery wall. This performance is called common shear stress [2, 14] and it would be increased by HTN [15]. Also, HTN has been suggested as a risk factor for CAE [10, 16–19]. HTN is a global problem, especially in developing countries [20]. Approximately, 16.5 % of deaths annually (9.5 million deaths) are attributed to HTN [21]. HTN is defined as systolic blood pressure (SBP) ≥ 140 mmHg and/or diastolic blood pressure (DBP) ≥ 90 mmHg according to the World Health Organization.

Although the association of HTN and CAE has been widely studied, information regarding the relationship between this factor and the CAE is limited. Many studies have not reported the adjusted effect of HTN on CAE [22–26]. It is important to investigate the adjusted effect of HTN on CAE because there are controversial results between available studies. A study reported 114 % more chance of CAE for subjects with HTN than the control group [27]. Another study showed 142 % more chance of CAE in the HTN group [28]. In contrast, a study expressed ineffective HTN on CAE [29] or the other study showed the prospective effect of HTN on CAE [30]. To the best of our knowledge, there is no meta-analysis study to show this relation.

Based on the literature review, the risk factors related to the CAE were quantitatively analyzed. Therefore, this systematic review and meta-analysis study aimed to clarify and quantify of HTN effect on CAE.

## Methods

### Data source and search strategy

This study was prepared according to the preferred reporting items for systematic reviews and meta-analyses (PRISMA) statement [31]. A comprehensive systematic search was conducted in electronic databases including Scopus, PubMed, and Science Direct without a time limit until July 25, 2019. We used the following keywords and Mesh (Medical Subject Headings) terms to search literature: (“coronary artery ectasia” OR “CAE” OR “coronary heart disease” OR “CAD” OR “coronary artery aneurysm” AND “hypertension” OR “HTN” OR “blood pressure”). We also checked the references of the obtained articles to find more relevant potential publications.

### Inclusion and exclusion criteria

Inclusion criteria were as follows: (1) articles with full text in English, (2) publications considered odds ratio (OR) with 95 % confidence interval (CI) for CAE between subjects with and without HTN, or (3) reported the number of subjects with and without HTN in CAE and non-CAE groups. Publications were excluded with the following characteristics: (1) duplicates, (2) books, case reports, conference, and editorial articles, (3) absence of HTN, (4) full text not available. The obtained articles were reviewed by two authors (EZ and MB) and confirm with the third person (MR).

### Data extraction and quality assessment

Information was collected for each included study: study ID (first author’s name), publication year, country that study conducted in, type of study (case-control and cross-sectional), participant characteristics (number of sample size and age), outcomes require characteristics (HTN, DM, family history of coronary artery disease [CAD], recently smoked, hyperlipidemia, and body mass index [BMI]). Before selecting studies to enter the meta-analysis, we assessed them for risk of bias. We used the Mixed Method Appraisal Tool (MMAT) (“quantitative non-randomized” part of MMAT) to investigate the quality of included studies. MMAT can be used for studies with different structures, including qualitative, quantitative, and mixed with different designs. The reliability and validity of MMAT were confirmed in previous studies [32]. This checklist is designed to have two basic questions that will be further explored if any study is accepted on both questions. The quality of articles is evaluated with five questions in the “quantitative non-randomized” part of MMAT. The quality of articles is determined by high quality (5), medium quality (4 or 3), or low quality (1 or 2). The summary results of the quality assessment were provided in Table 1.

**Table 1:** Characteristics of included studies

Study ID	Year	Country	CAE+	CAE–	Design	HTN	DM	F.CAD	Hyp	Smoke	BMI	MMAT score
Aghajani et al. [33]	2017	Iran	27	33	Case-control	✓	✗	✗	✗	✗	✗	4
Akturk et al. [34]	2018	Turkey	40	40	Case-control	✓	✗	✗	✗	✗	✗	3
Antonopoulos et al. [35]	2016	Greece	39	41	Case-control	✓	✗	✗	✗	✗	✗	2
Baysal et al. [36]	2018	Turkey	32	35	Cross-sectional	✓	✗	✗	✗	✗	✗	3
Boles et al. [30]	2017	Sweden	16	26	Cross-sectional	✓	✓	✓	✓	✓	✓	2
Brunetti et al. [37]	2014	Italy	14	15	Cross-sectional	✓	✗	✗	✗	✗	✗	2
Demir et al. [38]	2013	Turkey	126	122	Cross-sectional	✓	✗	✗	✗	✗	✗	3
Dogan et al. [39]	2016	Turkey	167	150	Cross-sectional	✓	✗	✗	✗	✗	✗	4
Dursun et al. [22]	2015	Turkey	30	30	Cross-sectional	✓	✓	✗	✗	✗	✓	3
Erdogan et al. [23]	2013	Turkey	49	30	Cross-sectional	✓	✓	✓	✓	✓	✓	4
Farrag et al. [24]	2013	Egypt	192	2,408	Cross-sectional	✓	✓	✓	✗	✓	✗	3
Gok et al. [25]	2018	Turkey	52	33	Case-control	✓	✓	✗	✗	✓	✗	5
Ipek et al. [26]	2016	Turkey	99	1,556	Case-control	✓	✓	✗	✓	✓	✗	4
Isik et al. [40]	2012	Turkey	75	96	Cross-sectional	✓	✓	✓	✗	✓	✗	5
Iwanczyk et al. [28]	2019	Poland	27	27	Cross-sectional	✓	✗	✗	✗	✗	✗	3
Kalaycioglu et al. [41]	2014	Turkey	138	139	Cross-sectional	✓	✓	✓	✓	✓	✗	5
Karaagac et al. [42]	2014	Turkey	28	22	Case-control	✓	✓	✗	✗	✓	✗	2
Katritsis et al. [16]	2010	France	27	30	Cross-sectional	✓	✓	✗	✗	✓	✓	3
Kiris et al. [17]	2012	Turkey	34	24	Cross-sectional	✓	✓	✓	✗	✓	✓	4
Kiziltunc et al. [43]	2016	Turkey	41	72	Cross-sectional	✓	✓	✗	✗	✓	✗	3
Kundi et al. [44]	2017	Turkey	52	33	Cross-sectional	✓	✗	✗	✗	✗	✗	3
Liang et al. [45]	2019	China	87	90	Cross-sectional	✓	✗	✗	✗	✗	✗	4
Liu et al. [46]	2016	China	32	31	Case-control	✓	✓	✓	✗	✓	✓	3
Luo et al. [47]	2017	China	51	100	Case-control	✓	✗	✗	✗	✗	✗	5
Ozbek et al. [5]	2016	Turkey	117	70	Cross-sectional	✓	✓	✓	✓	✓	✓	4
Ozde et al. [6]	2018	Turkey	55	55	Case-control	✓	✓	✓	✗	✓	✓	4
Qin et al. [7]	2019	China	100	100	Cross-sectional	✓	✓	✗	✗	✓	✓	3
Quisi et al. [8]	2018	Turkey	51	50	Case-control	✓	✓	✓	✓	✓	✓	4
Sarli et al. [18]	2014	Turkey	210	100	Case-control	✓	✗	✗	✗	✗	✗	5
Satiroglu et al. [48]	2015	Turkey	20	28	Cross-sectional	✓	✓	✓	✗	✓	✓	3
Schram et al. [27]	2018	Netherland	77	154	Case-control	✓	✗	✗	✗	✗	✗	2
Sen et al. [19]	2014	Turkey	100	80	Case-control	✓	✓	✓	✗	✓	✓	5
Sen et al. [49]	2007	Turkey	67	45	Cross-sectional	✓	✓	✗	✗	✓	✓	3
Tuzun et al. [29]	2007	Turkey	35	35	Cross-sectional	✓	✗	✗	✗	✗	✗	3
Uygun et al. [50]	2019	Turkey	41	45	Cross-sectional	✓	✓	✓	✗	✓	✓	5
Varol et al. [51]	2009	Turkey	366	160	Case-control	✓	✗	✗	✗	✗	✗	3
Wang et al. [52]	2017	China	72	72	Cross-sectional	✓	✗	✗	✗	✗	✗	5
Yalcin et al. [9]	2015	Turkey	40	44	Cross-sectional	✓	✓	✓	✗	✓	✓	4
Yang et al. [10]	2013	China	131	1,269	Cross-sectional	✓	✓	✓	✗	✓	✓	4
Yolcu et al. [11]	2016	Turkey	62	57	Cross-sectional	✓	✓	✓	✓	✓	✗	3

*CAE+* participants with coronary artery ectasia (CAE), *CAE–* participants without CAE; *HTN* hypertension (reported in study), *DM* diabetes mellitus (reported in study), *F.CAD* family history of coronary artery disease (reported in study), *Hyp* hyperlipidemia (reported in study); Smoke, recent smoked (reported in study), *BMI* body mass index (reported in study), *MMAT* Mixed Method Appraisal Tool

### Statistical analysis

In the present study, the random-effects model through a generic inverse-variance method was used to calculate the pooled OR and 95 % CI of CAE in subjects with HTN in comparison to subjects without HTN. The Stata/SE ver. 14 (Stata Corp., College Station, TX, USA) was used for meta-analysis and the following programs were: mean to conduct random-effects meta-analysis to obtain estimates for the relationship of HTN between CAE cases and the control group. Heterogeneity presented with calculated I^2^ index, and I^2^ values of 0 %, 25 %, 50 %, and 75 % represents no, low, moderate, and high heterogeneity, respectively [53]. We used funnel plots that announce publication bias that the Egger’s and begg’s tests confirm that by the statistical formula. A *P*-value of less than 0.05 was chosen to test the null hypothesis in all analyses [54, 55]. If heterogeneity exceeds 50 %, the Jackknife approach was used. The Jackknife approach examines the effect of each study. This method works by reporting the results of all other articles by excluding each study. If the result exceeds the specified CI, it indicates the high effect of that study. This method was used as a sensitivity analysis to estimate the potential publication bias on the overall estimates in the meta-analysis [56]. Also, subgroup analysis will distinct that there are significant differences in the type of study and quality of the study.

## Results

### Study selection

**Fig. 1:**
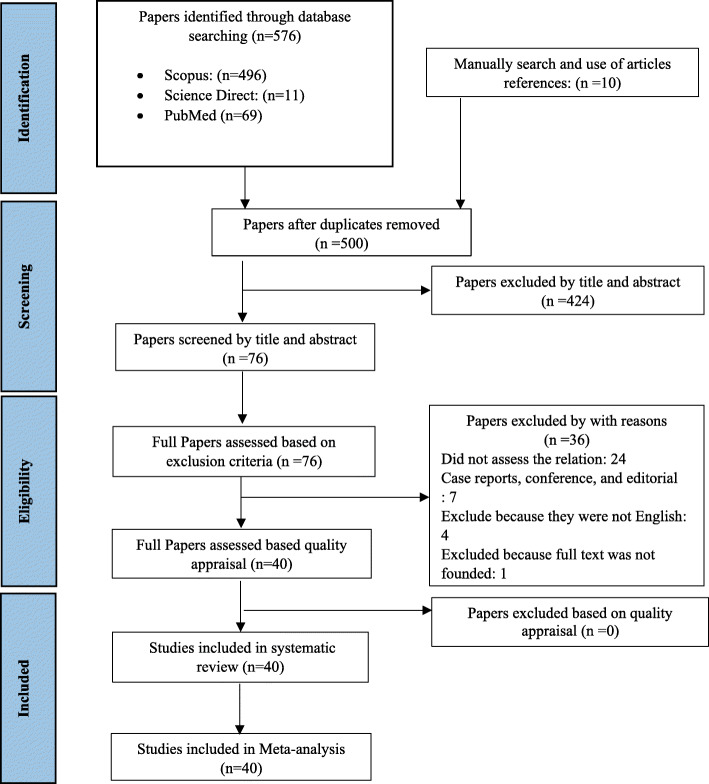
Inclusion criteria of preferred reporting items for systematic reviews and meta-analyses (PRISMA) flow chart

As shown in Fig. 1 and 576 studies were found based on the search strategy, and 10 studies by manually search. After deleting duplicate articles, 500 documents remained, and by examining their titles and abstracts, we reached 76 articles. After reviewing the full text of the remaining articles, 24 articles were discarded due to irrelevant results. Seven articles that were case reports, conferences, or editorials were also removed. Four papers were deleted for non-English text and one study was deleted due to lack of full text. Ultimately 40 articles [5–11, 16–19, 22–30, 33–52] were included in the meta-analysis.

### Study characteristics

The summary characteristics of studies were shown in Table 1, there are 40 studies [5–11, 16–19, 22–30, 33–52] include 14 case-control [6, 8, 18, 19, 25–27, 33–35, 42, 46, 47, 51] and 26 cross-sectional studies [5, 7, 9–11, 16, 17, 22–24, 28–30, 36–41, 43–45, 48–50, 52]. The studies with 11,047 participants, 3,263 cases, and 7,784 controls were included. All studies have determined the number of subjects with and without HTN in the two groups with and without CAE, which we can calculate the OR of CAE in the two groups. Only nine articles reported the adjusted OR of HTN allocated to CAE. The criteria for HTN were the same in all studies (SBP > 140 and/or DBP > 90). In all studies, men and women were examined simultaneously. In all studies, the mean age of participants was 47 to 71 years old in the case group (with CAE) and over 48 to 69 years old in the control group (without CAE). Studies have been done in different countries; there were six studies conducted in China, 28 in Turkey, and one for each of these countries (Egypt, France, Greece, Iran, Italy, Poland, Sweden, and The Netherlands). However, the criterion for assessing the outcome (CAE) is the same in all studies (coronary angiography), except for one study [24] that was performed with different criteria (scanner). The median year of publication of studies was 2016.

### Risk of bias

The obtained articles were reviewed by two authors and confirm with a third person. As mentioned in Table 1, the results examined for risk of bias for studies based on the MMAT, all studies had an acceptable quality for inclusion in the study and according to the “quantitative non-randomized” part of the checklist, eight studies had a low risk of bias [18, 19, 25, 40, 41, 47, 50, 52], 27 studies had a moderate risk of bias [5–11, 16, 17, 22–24, 26, 28, 29, 33, 34, 36, 38, 39, 43–46, 48, 49, 51], and five studies had a high risk of bias [27, 30, 35, 37, 42].

### Statistical analyses

**Fig. 2:**
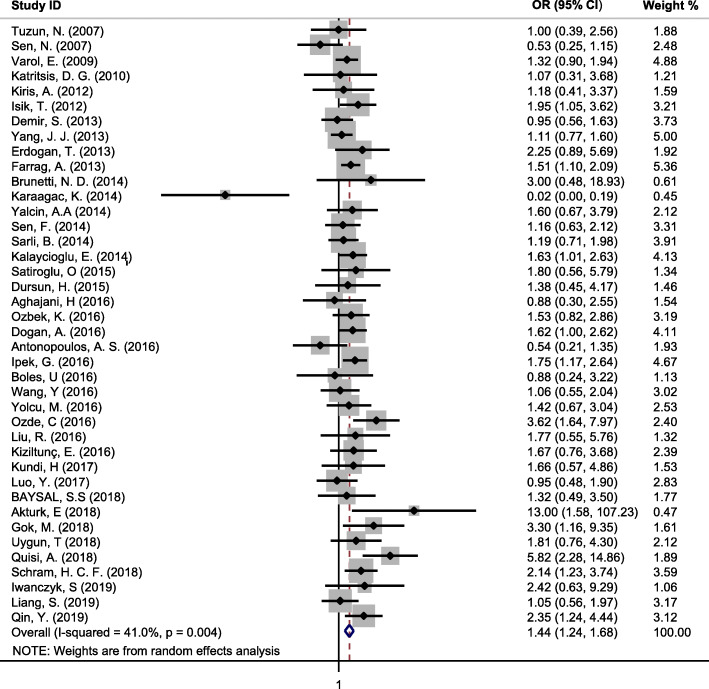
Forest plot of the association between hypertension and coronary artery ectasia in the unadjusted model. OR, odds ratio; CI, confidence interval

**Fig. 3:**
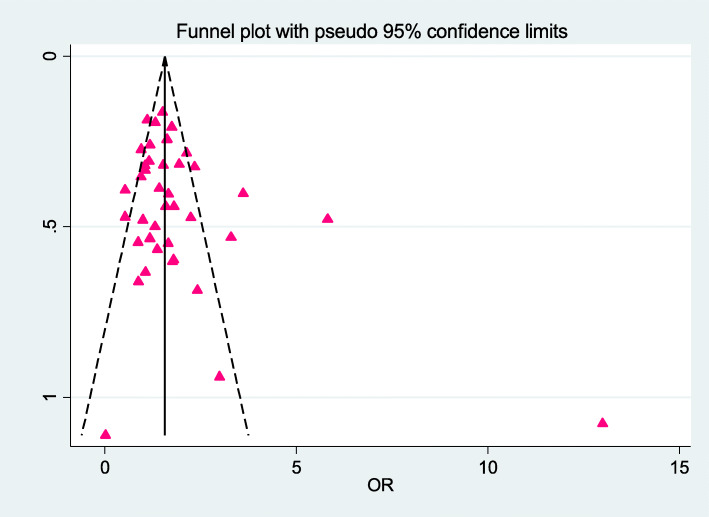
Funnel plot of hypertension and coronary artery ectasia of publication bias. OR, odds ratio

Forty studies with 3,263 cases and 7,784 controls that investigated the association between HTN and CAE were included. The pooled unadjusted OR of CAE in subjects with HTN compared to subjects without HTN was estimated 1.44 (95 % CI, 1.24 to 1.68) with low heterogeneity (I^2^ = 41 %; Cochran’s Q *P* = 0.004) (Fig. 2). There was no evidence of publication bias in the analysis of HTN and CAE with Egger’s test (*P* = 0.171), Begg’s test (*P* = 0.179), and study effect sizes distributed in a funnel plot (Fig. 3).

### Sensitivity and subgroup analyses

**Fig. 4:**
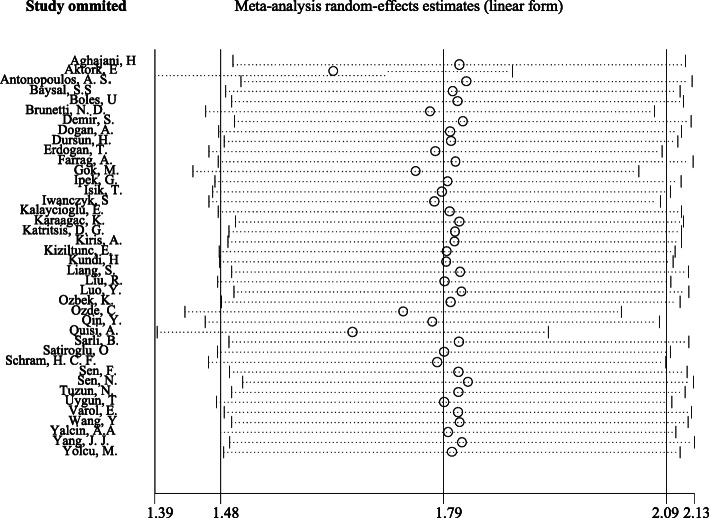
Sensitivity analysis plots using the Jackknife approach

The Jackknife method was used to investigate the effect of each study on the total effect size and heterogeneity. As shown in Fig. 4, none of the studies alone have a significant effect on the overall result of the study and do not distort the overall result, and it cannot be concluded that the heterogeneity that exists is due to the existence of a particular study. To further investigate the heterogeneous cause, we used subgroup analysis and examined the outcome for the type of study and quality of the study. For the type of study, results showed that in case-control studies, the OR for subjects with HTN compared to subjects without HTN was 1.53 (95 % CI, 1.06 to 2.21) with moderate heterogeneity (I^2^ = 71.3 %; Cochran’s Q *P* = 0.001). But in cross-sectional studies, the OR for subjects with HTN than subjects without HTN was 1.39 (95 % CI, 1.22 to 1.58) with no heterogeneity (I^2^ = 0 %; Cochran’s Q *P* = 0.738) (Fig. S1). For the quality of the study, based on the scoring of the MMAT, the studies were divided into three categories: high quality, moderate quality, and low quality. In high quality studies, the outcome (OR) was 1.40 (95 % CI, 1.12 to 1.76) with no heterogeneity (I^2^ = 1.7 %; Cochran’s Q *P* = 0.417). In moderate quality studies, the outcome was 1.49 (95 % CI, 1.27 to 1.76) with low heterogeneity (I^2^ = 29.7 %; Cochran’s Q *P* = 0.075), but in low quality studies, the outcome was 0.71 (95 % CI, 0.21 to 2.35) with high heterogeneity (I^2^ = 81.4 %; Cochran’s Q *P* = 0.001) (Fig. S2). Additionally, we utilized the meta-regression analysis for the year of publication of the articles. For one year of increasing articles, the possibility of increasing the risk of CAE in people with HTN was reported to be 18 % higher (95 % CI, 1.10 to 1.30) with high heterogeneity (I^2^ = 85.4 %; Cochran’s Q *P* < 0.001) (Fig. S3).

## Discussion

The main purpose of this systematic review and meta-analysis study was to evaluate the chances of CAE due to HTN. To find potentially relevant records, electronic databases were searched. Finally, 40 articles were included in the meta-analysis. The risk of CAE in subjects with HTN was 44 % higher than in subjects without HTN. Our result was consistent with a meta-analysis study that evaluated the risk of HTN on a similar disease (abdominal aortic aneurysms) that reported a 66 % higher risk of abdominal aortic aneurysms in high HTN patients [57]. The result of a recent study showed 135 % more chance of CAE in HTN subjects than the no-HTN group [7]. Also, a study reported 66 % more chance of CAE in HTN subjects than the control group [44] that confirms the effect of HTN on CAE.

One of the clinical reasons for CAE might be the pressure effect on the artery wall in the blood flow. This is because HTN is caused by two parts, the systole, and the diastole. By pumping blood through the arteries from the heart (systole) and returning blood from the arteries to the heart (diastole), it results in the artery dilate by pushing blood on the artery wall. This performance is called common shear stress [2, 14]. But we are looking for the adjusted effect of HTN on CAE. Because HTN is caused by unhealthy lifestyles, it needed to clarify whether this effect on CAE is directly related to HTN or not.

Variety factors could confuse the HTN effect on CAE, for instance, BMI is an important factor that affected several diseases. A person with a BMI above 25 usually has high blood lipids (hyperlipidemia) [58, 59]. This disease is usually chronic and requires ongoing medication to control blood lipid levels [2]. Blood lipids over time cause fat to build up in the arteries and it makes the arteries narrow. Narrowing in one part of the artery can put too much pressure on the artery and dilate the area around it [60, 61]. This confirmed by our results, it was a 35 % higher chance of CAE in hyperlipidemia cases than in the control group (Fig. S4).

DM is the factor that has a potential effect on coroners. In DM, the destruction of beta-cell occurs in the pancreas [62]. The main cause of the loss of β cells is cellular damage caused by the cellular immune response [63]. Following this destruction, markers are released into the bloodstream, causing damage to the vein [64]. This is in line with the results we achieved. We found that DM increased a 19 % chance of CAE than the control group (Fig. S5).

Smoking is one of the main causes of cardiovascular disease [65, 66]. By smoking, carbon monoxide and other toxic substances enter the bloodstream, which reduces blood oxygenation and less oxygen to the heart, which increases heart pumping and increases blood pressure that leads to a contraction in the arteries, this condition is a risk factor for CAE [67–70]. Our analysis found that those who had recently smoked were in a 53 % more chance of developing the CAE than the control group (Fig. S6).

Some diseases can be influenced by genetic or familial factors. Cardiovascular disease is one of these diseases that family history can increase the risk of that [71–73]. In our research, we found that there was a 22 % higher chance of CAE in family history of CAD cases than in the control group (Fig. S7).

**Fig. 5:**
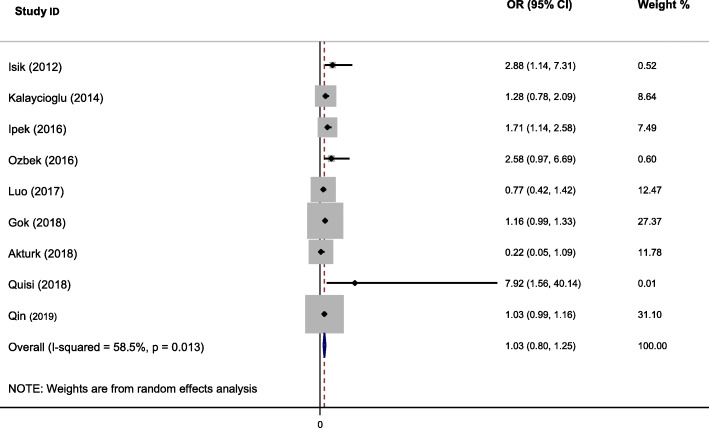
Forest plot of the association between hypertension and coronary artery ectasia in the adjusted model. OR, odds ratio; CI, confidence interval

The presence of different variables along with HTN can confound the results of the study. For investigating the adjusted effect of HTN on CAE, we survey adjusted reports of HTN. In the 40 articles reviewed, nine articles reported the adjusted effect of HTN on CAE by 624 cases and 628 controls [5, 7, 8, 25, 26, 34, 40, 41, 47]. The findings indicated the overall adjusted OR was 1.03 (95 % CI, 0.80 to 1.25) with moderate heterogeneity (I^2^ = 58.5 %; Cochran’s Q *P* = 0.013) (Fig. 5). This states a 41 % lower effect of unadjusted effect (OR = 1.44), which indicates the interaction rule of other factors.

### Limitations

It’s should be noted that this study was accompanied by several limitations both in study and outcome levels. The included studies had case-control and cross-sectional designs that are associated with inherent limitations to investigate a cause-effect relationship, for this limitation we performed subgroup analysis. Case-control studies show a stronger effect between HTN and CAE, but as you can see, there is higher heterogeneity in these studies (Fig. S1). This is because CAE is a chronic disease and the effect of variables on this disease is time-consuming.

Besides, the studies were carried out in different countries and it can be discussed whether the results of these studies can be combined. A review of the studies revealed that all studies except one of them used a measurement criterion to diagnose the disease. Based on MMAT, articles had different qualities. To investigate this issue, we used the subgroup analysis. Eight studies a had high quality that OR for CAE in HTN subjects than Non-HTN subjects was 1.40 (95 % CI, 1.12 to 1.76) with no heterogeneity (I^2^ = 1.7 %; Cochran’s Q *P* = 0.417), 27 studies had a moderate quality that OR was 1.49 (95 % CI, 1.27 to 1.76) with low heterogeneity (I^2^ = 29.7 %; Cochran’s Q *P* = 0.075) and five studies had a low quality that OR between two groups was 0.71 (95 % CI, 0.21 to 2.35) with high heterogeneity (I^2^ = 81.4 %; Cochran’s Q *P* = 0.001) (Fig. S2). After eliminating the low-quality studies, the results were as follows. OR between the two groups was 1.46 (95 % CI, 1.28 to 1.67) with low heterogeneity (I^2^ = 23.0 %; Cochran’s Q *P* = 0.113) (Fig. S8). It was better to examine this outcome for the sex variable, but none of the studies examined the effect of HTN on CAE for the sex variable. Also, the number of participants in the two groups of subjects (with HTN and without HTN), was not reported separately and we could not calculate the OR for sex effect on CAE. It is suggested that this outcome for the sex variable be investigated in future studies.

## Conclusions

We found that when the vessel was in normal condition, HTN was not very effective in increasing the chance of CAE. When a person had other risk factors which caused the vessels to be abnormal, the HTN increased the chance of CAE 44 %, while the adjusted HTN effect only increased the CAE chance by 3 %. More longitudinal studies are needed to more accurately investigate the effect of HTN on CAE by considering the limitations mentioned.

## Supplementary Information

**Additional file 1: Figure S1.** Forest plot of the association between type of study and coronary artery ectasia.

**Additional file 2: Figure S2.** Forest plot of the association between quality of study and coronary artery ectasia.

**Additional file 3: Figure S3.** Bubble plot for the effect of year on the coronary artery ectasia by meta-regression analysis.

**Additional file 4: Figure S4.** Forest plot of the association between hyperlipidemia and coronary artery ectasia.

**Additional file 5: Figure S5.** Forest plot of the association between diabetes mellitus and coronary artery ectasia.

**Additional file 6: Figure S6.** Forest plot of the association between recently smoked and coronary artery ectasia.

**Additional file 7: Figure S7.** Forest plot of the association between family history of heart disease and coronary artery ectasia.

**Additional file 8: Figure S8.** Forest plot of the association between high and moderate studies and coronary artery ectasia.

## Data Availability

All data that support the conclusions of this manuscript are included within the article [5–11, 16–19, 22–30, 33–52].

## Abbreviations

BMI: Body mass index
CAD: Coronary artery disease
CAE: Coronary artery ectasia
CI: Confidence interval
DBP: Diastolic blood pressure
DM: Diabetes mellitus
HTN: Hypertension
MMAT: Mixed Method Appraisal Tool
OR: Odds ratio
PRISMA: Preferred Reporting Items for Systematic Reviews and Meta-Analyses
SBP: Systolic blood pressure
