# Supplementary figures and images for: Hypertension and coronary artery ectasia: a systematic review and meta-analysis study
Source: Clin Hypertens. 2021 Jul 15;27:14. doi: 10.1186/s40885-021-00170-6 (PMC8281588; doi:10.1186/s40885-021-00170-6)

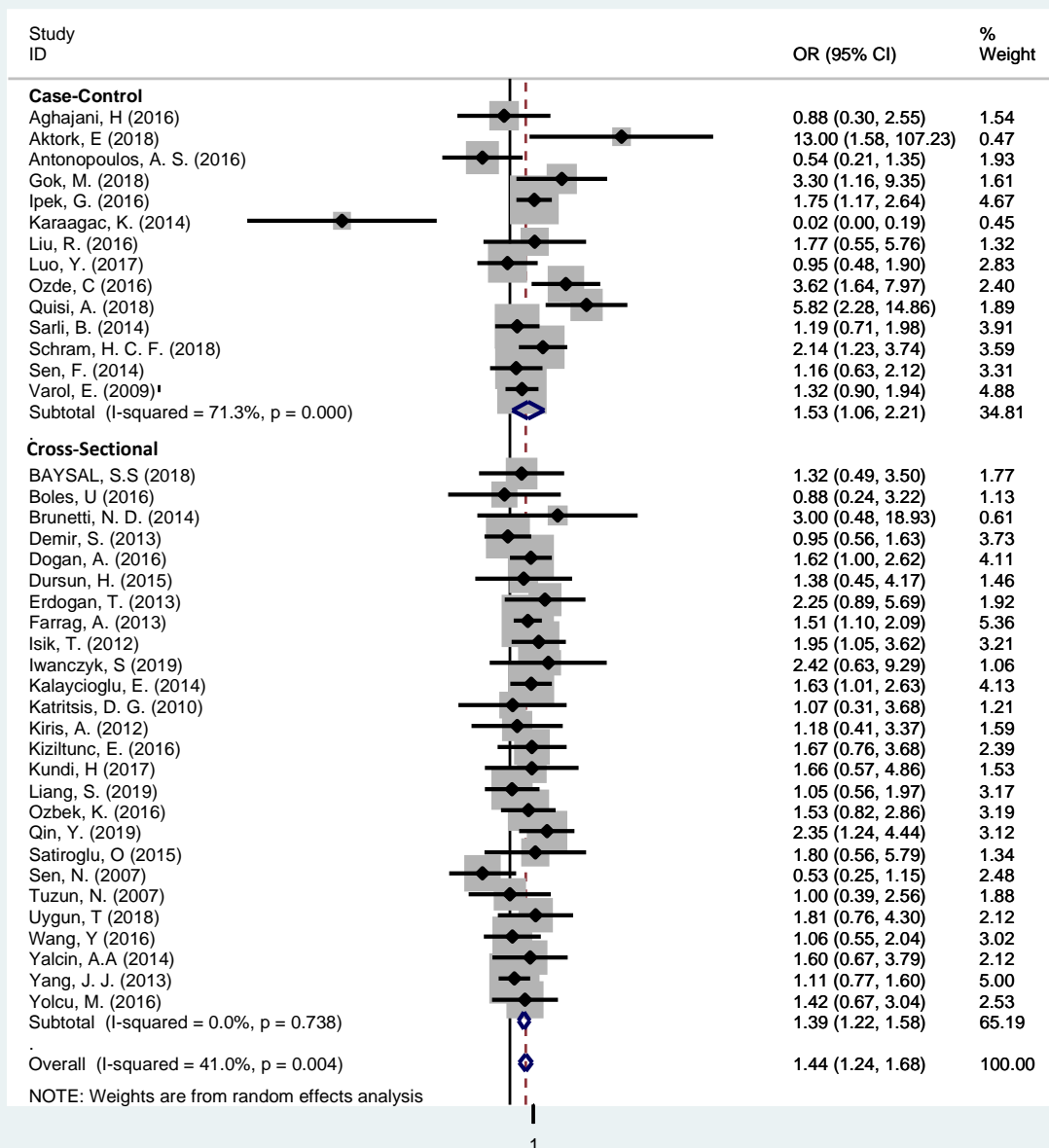

S. Figure 1. Forest plot of the association between type of study and CAE

Supplement: Supplementary file 1 — Additional file 1: Figure S1. Forest plot of the association between type of study and coronary artery ectasia. [file 40885_2021_170_MOESM1_ESM.pdf]

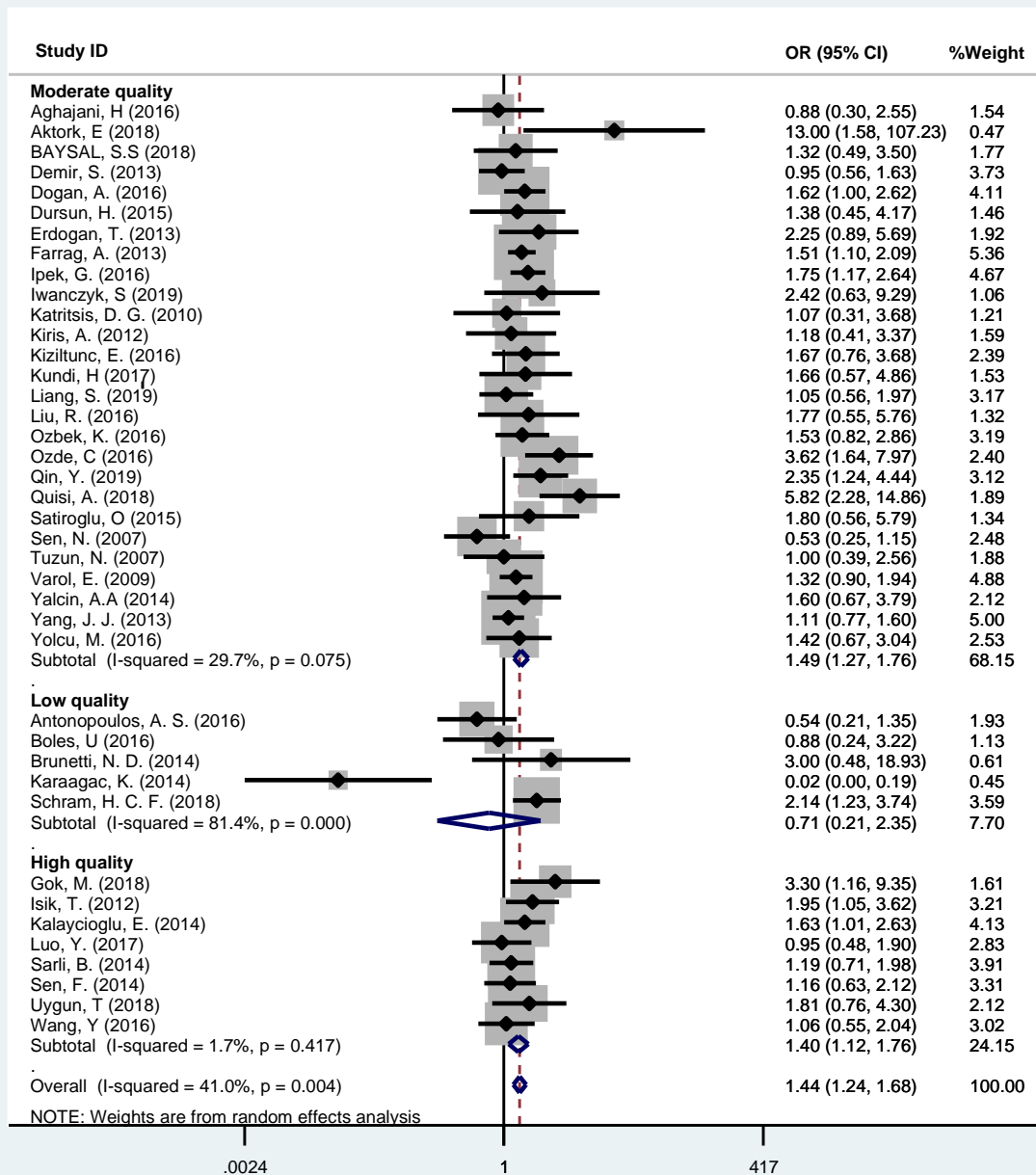

S. Figure 2. Forest plot of the association between quality of study and CAE

Supplement: Supplementary file 2 — Additional file 2: Figure S2. Forest plot of the association between quality of study and coronary artery ectasia. [file 40885_2021_170_MOESM2_ESM.pdf]

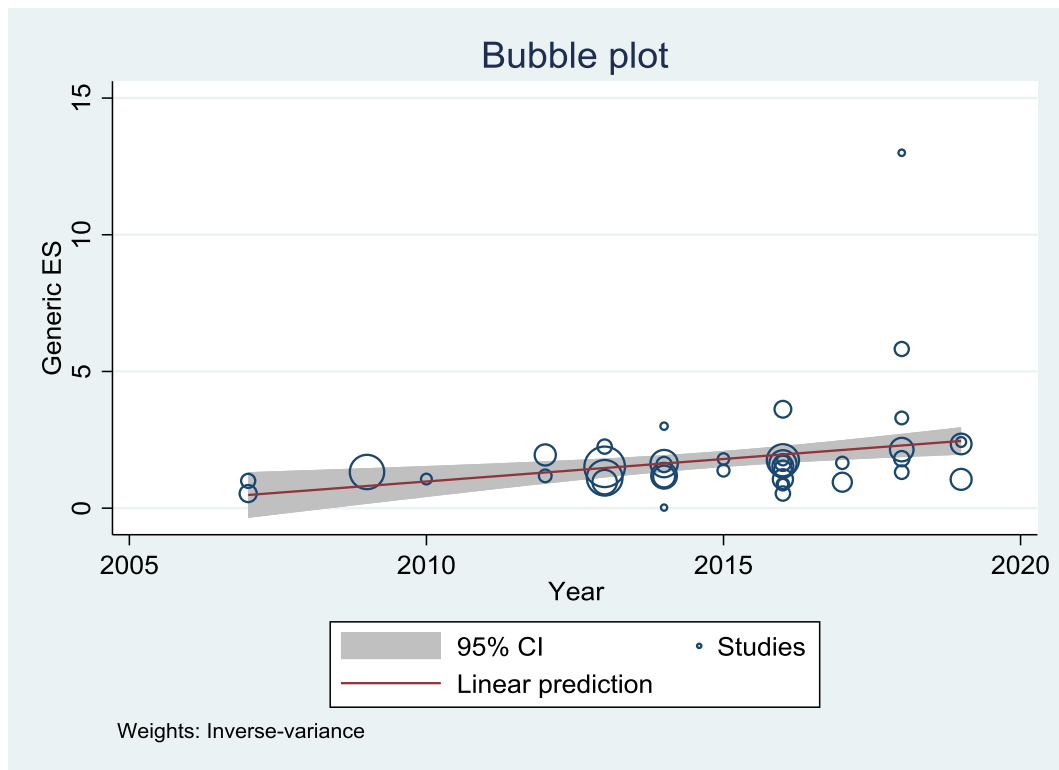

S. Figure 3. Bubble plot for the effect of year on the CAE by meta-regression analysis

Supplement: Supplementary file 3 — Additional file 3: Figure S3. Bubble plot for the effect of year on the coronary artery ectasia by meta-regression analysis. [file 40885_2021_170_MOESM3_ESM.pdf]

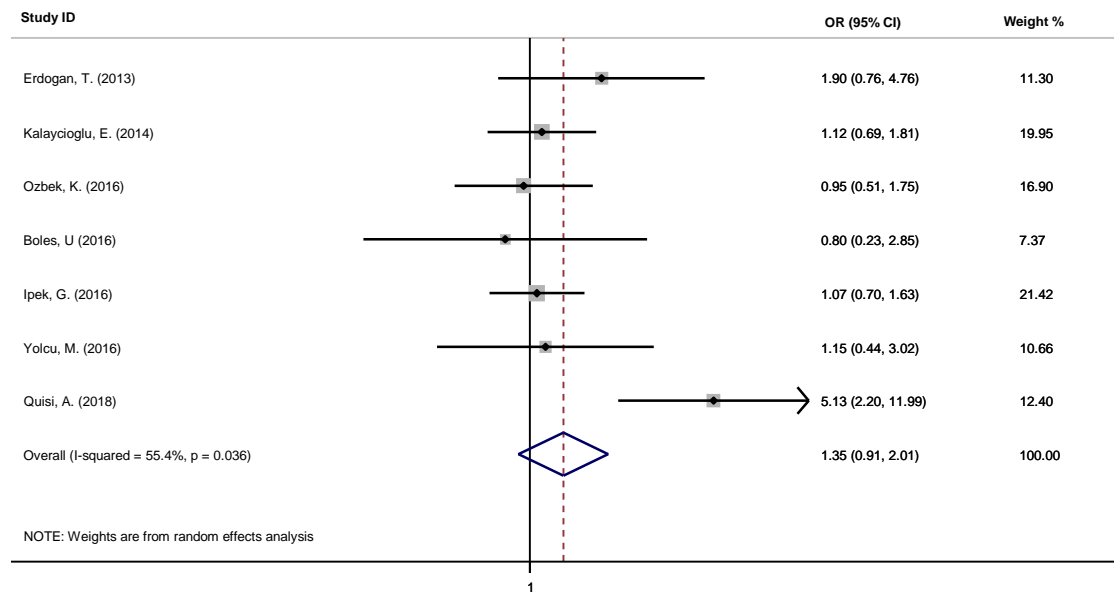

S. Figure 4. Forest plot of the association between hyperlipidemia and CAE

Supplement: Supplementary file 4 — Additional file 4: Figure S4. Forest plot of the association between hyperlipidemia and coronary artery ectasia. [file 40885_2021_170_MOESM4_ESM.pdf]

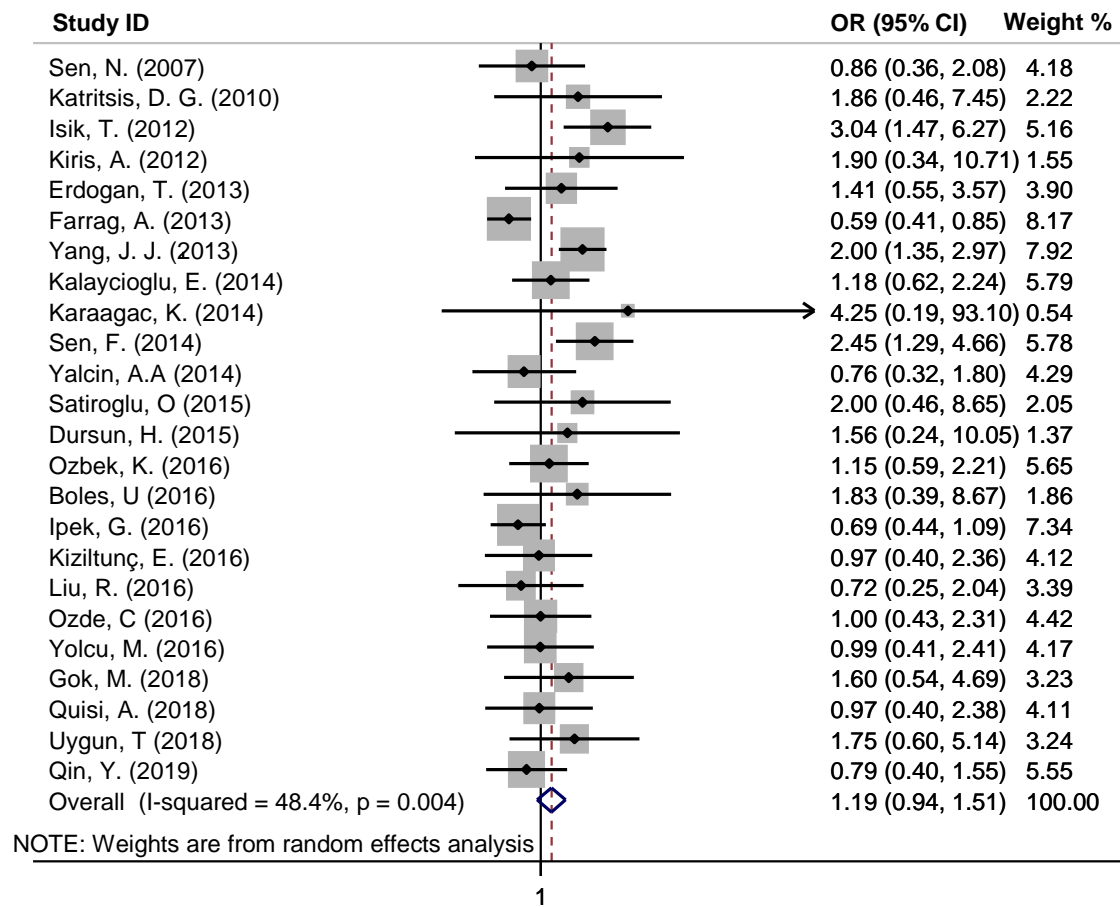

S. Figure 5. Forest plot of the association between DM and CAE

Supplement: Supplementary file 5 — Additional file 5: Figure S5. Forest plot of the association between diabetes mellitus and coronary artery ectasia. [file 40885_2021_170_MOESM5_ESM.pdf]

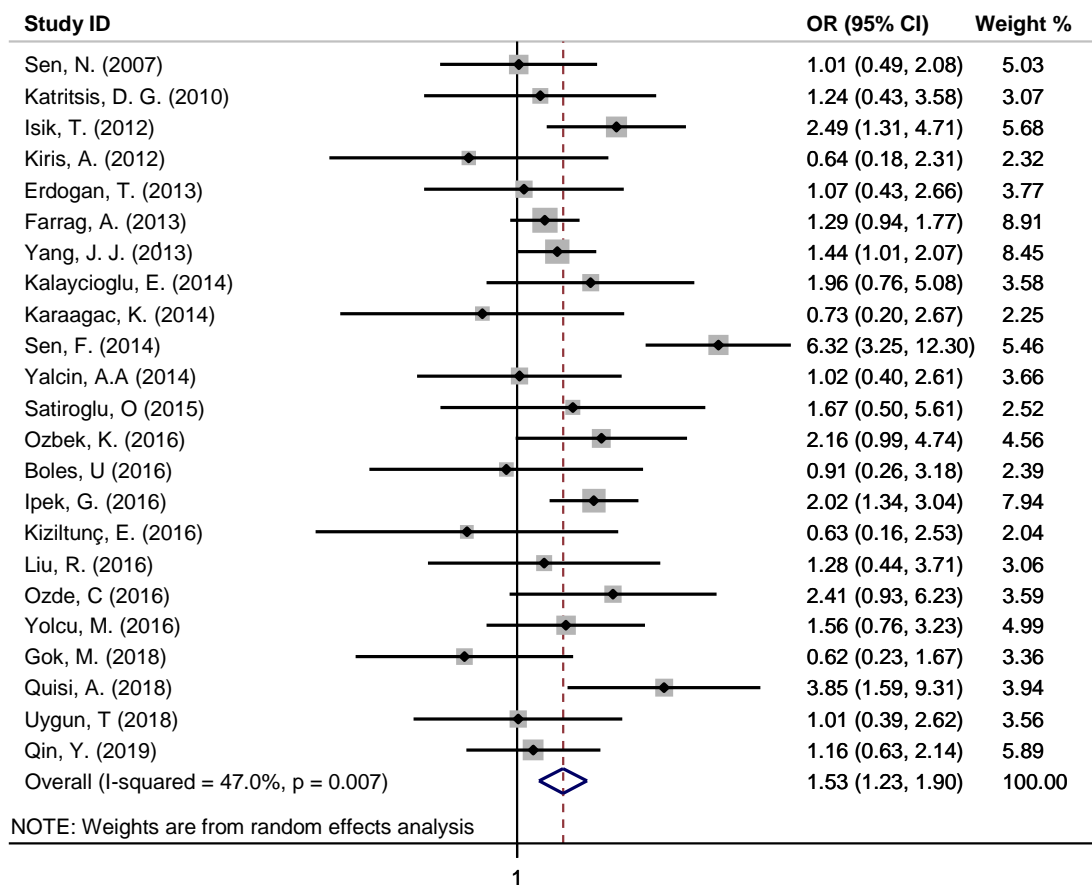

S. Figure 6. Forest plot of the association between recently smoked and CAE

Supplement: Supplementary file 6 — Additional file 6: Figure S6. Forest plot of the association between recently smoked and coronary artery ectasia. [file 40885_2021_170_MOESM6_ESM.pdf]

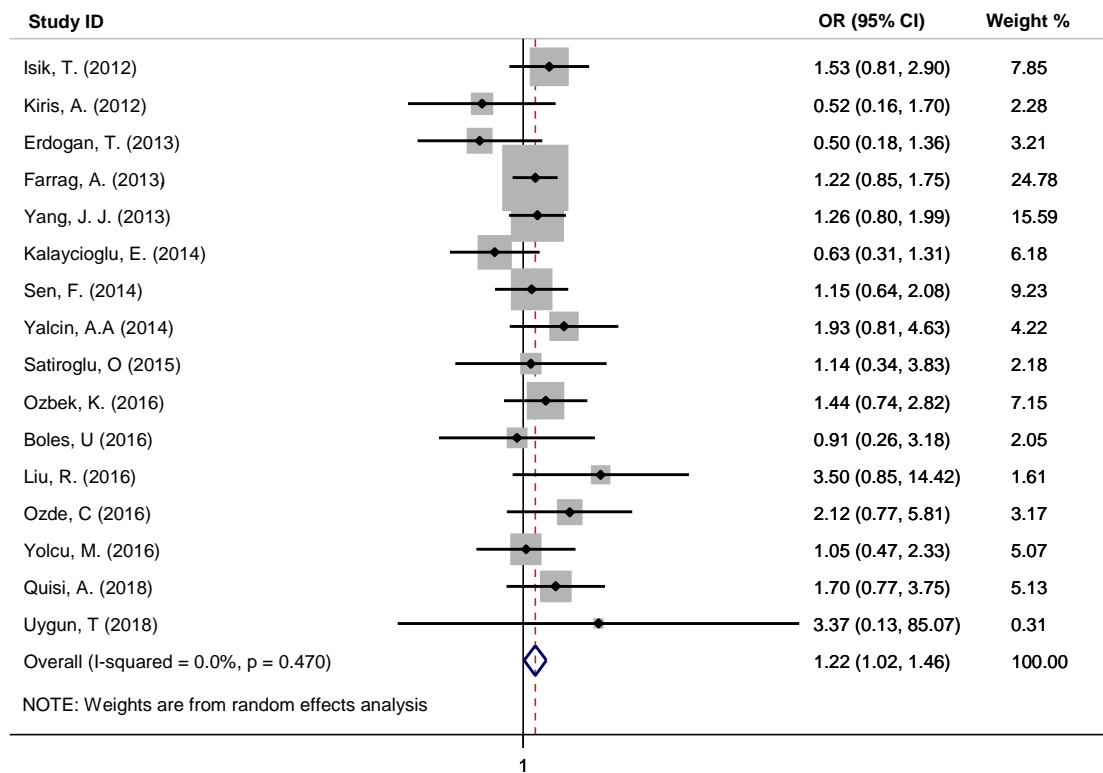

S. Figure 7. Forest plot of the association between family history of heart disease and CAE

Supplement: Supplementary file 7 — Additional file 7: Figure S7. Forest plot of the association between family history of heart disease and coronary artery ectasia. [file 40885_2021_170_MOESM7_ESM.pdf]

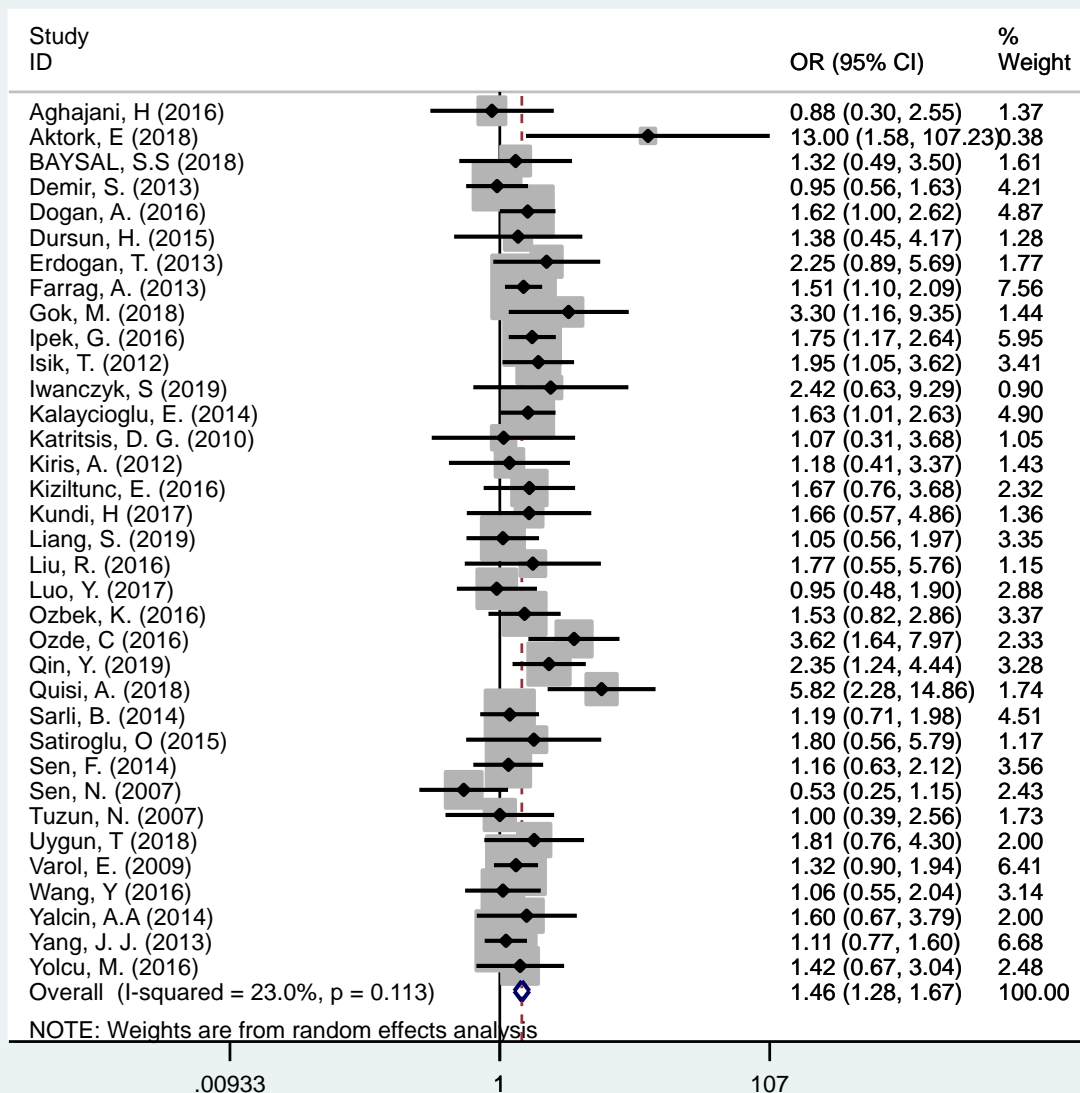

S. Figure 8. Forest plot of the association between high and moderate studies and CAE

Supplement: Supplementary file 8 — Additional file 8: Figure S8. Forest plot of the association between high and moderate studies and coronary artery ectasia. [file 40885_2021_170_MOESM8_ESM.pdf]
